# Supplementary material for: Investigating the Relation of Intelligence and Executive Functions in Children and Adolescents with and without Intellectual Disabilities
Source: Children (Basel). 2022 Jun 1;9(6):818. doi: 10.3390/children9060818 (PMC9221765; doi:10.3390/children9060818)
Supplement: Supplementary file 1 [file children-09-00818-s001.zip › children-1670567-supplementary.pdf]

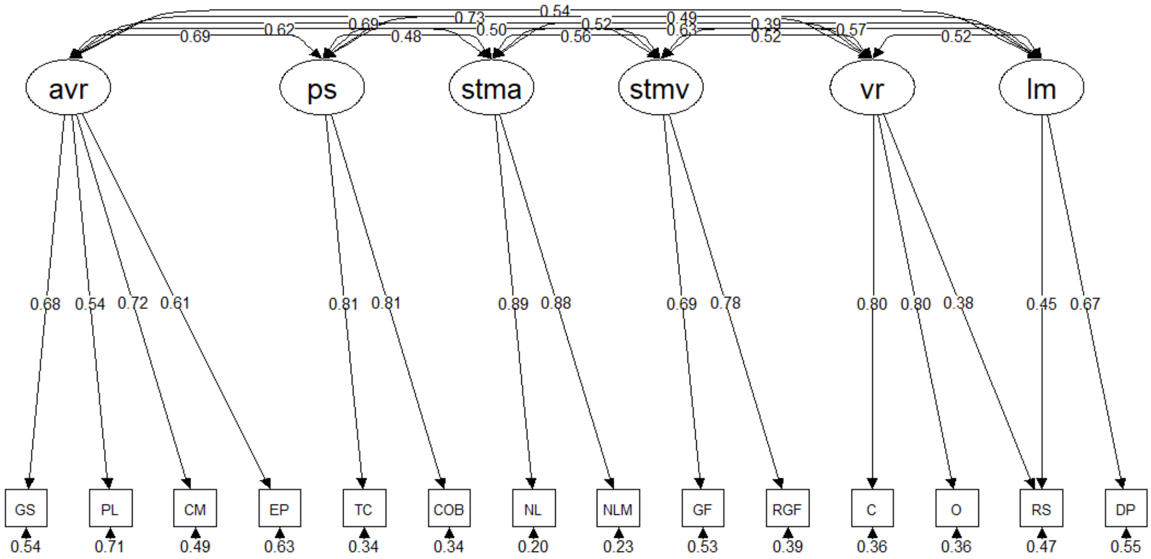

Figure S1. Confirmatory factor analysis of the intelligence subtests included in the Intelligence and Development Scales [22]. Latent variables are represented by ovals; manifest indicators are depicted as squares. avr = *Abstract Visual Reasoning*, ps = *Processing Speed*, stma = *Auditive Short-Term Memory*, stmv = *Visuo–Spatial Short-Term Memory*, vr = *Verbal Reasoning*, lm = *Long-Term Memory*, GS = “Geometric Shapes”, PL = “Plates”, CM = “Completing Matrices”, EP = “Excluding Pictures” ,TC = “Two Characteristics”, COB = “Crossing-Out Boxes”, NL = “Numbers/Letters”, NLM = “Numbers/Letters Mixed”, GF = “Geometric Figures”, RGF = “Rotated Geometric Figures”, C = “Categories”, O = “Opposites”, RS = “Retelling a Story”, DP = “Describing a Picture”.

Table S1. Absolute and relative frequencies of demographic characteristics

Characteristic	Full sample (*N* = 1540)		BID subsample (*n* = 255)	
Gender				
Male	738	(47.92)	143	(56.1)
Female	802	(52.08)	112	(43.9)
Age				
8	163	(10.6)	61	(10.2)
9	153	(9.9)	62	(7.8)
10	142	(9.2)	64	(10.6)
11	145	(9.4)	51	(9.4)
12	119	(7.7)	49	(7.5)
13	102	(6.6)	37	(10.2)
14	133	(8.6)	51	(8.2)
15	120	(7.8)	53	(7.5)
16	93	(6.0)	31	(7.1)
17	87	(5.6)	42	(5.5)
18	79	(5.1)	35	(3.5)
19	101	(6.6)	30	(5.9)
20	103	(6.7)	32	(6.7)
Residency				
Swiss	820	(53.2)	91	(35.7)
German	649	(42.1)	155	(60.8)
Others	71	(4.6)	9	(3.5)
Educational status (mother)				
Compulsory education	174	(11.6)	57	(34.7)
Upper-secondary vocational training	561	(37.3)	261	(36.3)
Upper-secondary general education	91	(6.0)	34	(5.2)
Higher vocational training	124	(8.2)	54	(4.4)
Universities	531	(35.3)	171	(15.1)
Unknown	25	(1.7)	9	(4.4)

Note. BID = borderline intellectual disability (IQ ≤ 85). Percental frequencies are rounded to one decimal digit and are presented in brackets. Educational status groups according to Swiss Bundesamt für Statistik [22].

Table S2. Descriptive statistics of the indicators within the full sample (N = 1540)

	M	*SD*	**min**	**max**	**skew**	**kurtosis**
GS	10.08	3.208	1	19	-0.079	0.325
PL	10.08	3.130	1	19	-0.145	0.317
RS	10.08	3.118	1	19	-0.375	0.467
DP	10.30	3.104	1	19	0.136	0.310
TC	9.94	3.056	1	19	-0.234	0.983
COB	9.95	3.135	1	19	-0.082	0.691
NL	10.38	3.243	1	19	-0.195	0.509
NLM	10.39	3.093	1	19	-0.251	0.799
GF	10.14	3.047	1	19	0.533	0.522
RGF	10.11	3.162	1	19	0.251	0.884
CM	10.25	3.194	1	19	0.029	-0.104
EP	10.20	3.174	1	19	-0.116	0.249
C	10.37	3.256	1	19	-0.388	0.488
O	10.31	3.061	1	19	-0.352	0.665
LW	9.83	3.147	1	19	-0.096	-0.012
AC	9.89	3.090	1	19	-0.152	0.157
DA	9.94	2.494	1	18	-0.413	0.701
DR	10.10	2.567	1	17	-0.337	0.052

Note. GS = "Geometric Shapes", PL = "Plates", RS = "Retelling a Story", DP = "Describing a Picture", TC = "Two Characteristics", COB = "Crossing-Out Boxes", NL = "Numbers/Letters", NLM = "Numbers/Letters Mixed", GF = "Geometric Figures", RGF = "Rotated Geometric Figures", CM = "Completing Matrices", EP = "Excluding Pictures", C = "Categories", O = "Opposites", LW = "Listing Words", AC = "Animal Colors", DA = "Divide Attention", DR = "Drawing Routes".

Table S3. Factor loadings of the exploratory factor analysis of the executive function tasks

	**PA1**	**h^2**	**u**
LW	0.746	0.556	0.44
AC	0.517	0.267	0.73
DA	0.905	0.819	0.18
DR	0.299	0.089	0.91

Note: The principal axis approach was used for the exploratory factor analysis. Loadings above 0.3 are in bold face. PA1 = first principal axis extracted, h2 = communality, u = uniqueness. LW = “Listing Words”, AC = “Animal Colors”, DA = “Divide Attention”, DR = “Drawing Routes”.

Table S4. Factor loadings of the exploratory factor analysis of the executive function tasks within the (borderline) intellectual disability subsample, excluding multivariate outliers (*n* = 220)

	**PA1**	**h^2**	**u**
LW	0.738	0.545	0.455
AC	0.574	0.329	0.671
DA	0.971	0.943	0.057
DR	0.428	0.183	0.817

Note: The principal axis approach was used for the exploratory factor analysis. PA1 = first principal axis extracted, h2 = communality, u = uniqueness. LW = "Listing Words", AC = "Animal Colors", DA = "Divide Attention", DR = "Drawing Routes".

Table S5. Explorative analyses: maximum likelihood estimation and model fit statistics within the (borderline) intellectual disability subsample, excluding multivariate outliers (*n* = 220).

**Model**	Fit indices						
	χ^2	*df*	**CFI**	**RMSEA**	**SRMR**	**AIC**	**BIC**
Measurement models							
Intelligence	98.58	67	0.974	0.045	0.037	7003.44	7179.91
EF	2.47	2	0.999	0.030	0.019	2227.27	2268.00
Main analyses							
M1	169.80	120	0.973	0.040	0.044	10754.34	10998.69
M2	224.36	129	0.948	0.054	0.054	10792.14	11004.62
M2a	436.296	130	0.830	0.097	0.206	11009.77	11218.70
M2b	255.01	130	0.931	0.062	0.092	10822.70	11031.63

Note: The variances of the latent variables were constrained to unity to ensure model identification. Intelligence measurement model including five broad abilities in accordance with Grieder and Grob [84]. M1 = first-order CFA including six intelligence factors and one EF factor, M2 = second-order CFA: M1 additionally including the superordinate g-factor, M2a = M2 with the loading of g onto the EF factor fixed to 0, M2b = M2 with the loading of g onto the EF factor fixed to 1. CFI = comparative fit index, RMSEA = root mean square error of approximation, CI = 90% confidence interval for RMSEA, SRMR = standardized root mean square residual, AIC = Akaike's information criterion, BIC = Bayesian information criterion.
